# Treatable Traits for Asthma Management in Pregnancy (TTAP): protocol for an Australian multicentre prospective observational cohort study

**DOI:** 10.1136/bmjopen-2025-115626

**Published:** 2026-06-02

**Authors:** Jane E Grehan, Janet Bristow, Kelly Steel, Bronwyn K Brew, Michael J Peek, Annelies L Robijn, Helen L Barrett, Sean Seeho, Penelope Fotheringham, Marjorie Atchan, Soriah M Harvey, Sameh R N Samuel, Vanessa M McDonald, Megan E Jensen, Elizabeth G Holliday, Megan Rees, Elissa Elvidge, Lesley Vining, Kathleen Chapman, Esha Joshi, Andrew C Jones, Rebekah Bowman, Christopher J Brereton, Sarah AL Price, Kelly A McNamara, Amanda Beech, Craig E Pennell, Vanessa E Murphy

**Affiliations:** 1 College of Health Medicine and Wellbeing, The University of Newcastle, Callaghan, New South Wales, Australia; 2 Hunter Medical Research Institute, New Lambton Heights, New South Wales, Australia; 3 Reproduction and Perinatal Centre, The University of Sydney, Sydney, New South Wales, Australia; 4 Nepean Hospital, Penrith, New South Wales, Australia; 5 Sydney Adventist Hospital, Wahroonga, New South Wales, Australia; 6 University of New South Wales, Sydney, New South Wales, Australia; 7 The Royal Hospital for Women, Randwick, New South Wales, Australia; 8 Royal North Shore Hospital, St Leonards, New South Wales, Australia; 9 University of Canberra, Canberra, Australian Capital Territory, Australia; 10 Wollongong Hospital, Wollongong, New South Wales, Australia; 11 University of Wollongong, Wollongong, New South Wales, Australia; 12 The University of Melbourne, Melbourne, Victoria, Australia; 13 The Royal Melbourne Hospital, Melbourne, Victoria, Australia; 14 Maitland Hospital, Maitland, New South Wales, Australia; 15 Queanbeyan Hospital and Health Service, Queanbeyan, New South Wales, Australia; 16 The Royal Women’s Hospital, Parkville, Victoria, Australia; 17 Central Coast Local Health District, Gosford, New South Wales, Australia; 18 John Hunter Hospital, New Lambton Heights, New South Wales, Australia

**Keywords:** Asthma, Pregnancy, Prospective studies, Pregnant Women

## Abstract

**Abstract:**

**Introduction:**

Asthma is one of the most prevalent long-term health conditions affecting pregnant women. Poorly controlled asthma during pregnancy is associated with adverse maternal and fetal outcomes and may predispose offspring to long-term respiratory morbidity. The current ‘one size fits all’ approach to asthma management during pregnancy is not optimally effective for approximately half of the pregnant women with asthma. A personalised medicine approach to managing airways disease is required. The treatable traits approach focuses on the identification and treatment of traits in the pulmonary, extra-pulmonary and behavioural domains, which are identifiable, measurable, clinically relevant (linked to exacerbation risk or poor asthma control) and treatable. This manuscript outlines the protocol for the Treatable Traits for Asthma Management in Pregnancy (TTAP) study. The purpose of the TTAP study is to prospectively determine the prevalence of a range of treatable traits from these three domains in pregnant women with asthma and determine which traits are associated with exacerbation risk, poor asthma control and poor asthma-related quality of life. Additionally, this study will assess differences in trait prevalence and clinical relevance in pregnant women from regional versus metropolitan hospitals in Australia and in different antenatal models of care.

**Methods and analysis:**

The TTAP study is a multicentre, prospective observational cohort study. Study participants are pregnant women with asthma attending antenatal clinics at 10 metropolitan and regional hospitals (public and private) in NSW and Victoria, Australia. Assessment of traits from the pulmonary, extrapulmonary and behavioural domains as well as asthma outcomes is conducted at three gestational timepoints: 12–16 weeks, 22–26 weeks and 32–36 weeks of pregnancy. A follow-up assessment of asthma outcomes is conducted at 2–4 weeks postpartum. The outcomes assessed are asthma exacerbations requiring medical intervention (primary outcome), asthma symptom control and asthma-related quality of life. Traits and outcomes will be assessed using questionnaires, direct questioning, measurement of biomarkers, physical measurements and assessment of routinely collected data from medical records.

**Ethics and dissemination:**

The Hunter New England Human Ethics Committee (2024/ETH01289) has approved the TTAP study protocol. Outcomes will be published in peer-reviewed journals, presented at scientific conferences and disseminated online to participants, clinicians and other pregnant women with asthma and their families via the Asthma in Pregnancy Toolkit website https://asthmapregnancytoolkit.org.au/.

**STRENGTHS AND LIMITATIONS OF THIS STUDY**

- Multidimensional assessment will provide an understanding of the interaction between treatable traits and their association with asthma outcomes, with objective biomarkers offering additional insight beyond symptoms alone. This will guide future research towards tailored management strategies based on individual profiles, promoting more effective and person-centred care.
- Inclusion of both regional and metropolitan (public and private) settings may improve the applicability of study findings to a broader population of pregnant women with asthma.
- Reflective of real-world clinical practice and consumer experiences, providing insights that are directly applicable to antenatal care.
- May be subject to volunteer selection bias, as women who consent to participate may differ from those who decline (eg, in asthma severity or health literacy) and from those not reached through recruitment—such as those accessing alternative antenatal care pathways—potentially affecting generalisability.
- Causality cannot be established using this observational design; findings can only be interpreted as associations; we acknowledge that there may be some residual selection and collider bias.

## Introduction

 Asthma is one of the most prevalent long-term health conditions among pregnant women, affecting up to 17% of pregnancies worldwide.^1^ Australia has one of the highest rates in the world, with asthma affecting 12%–17% of pregnancies.^1 2^ Pregnancy is a unique time when the impacts of the mother’s health extend beyond the mother to the baby,^3^ making it an important window of opportunity to influence the trajectory of health for the next generation.

Pregnant women with asthma have an increased risk of poor perinatal outcomes such as low birth weight, preterm birth and neonatal hospitalisation.^4^,^7^ Additionally, children born to mothers with asthma are at three times the risk of developing asthma compared with children whose mothers do not have asthma.^8^ This is greater than the risk in children whose fathers have asthma, strongly suggesting a role for the pregnancy environment in determining future asthma risk beyond genetics and the psychosocial environment. Recent studies show that around 40% of women with asthma experience a worsening of symptoms during pregnancy^8^ and 45% experience exacerbations requiring medical intervention.^9^ Poor asthma symptom control and exacerbations during pregnancy have been shown to further increase the risk of adverse perinatal outcomes^10^ and lead to worse respiratory outcomes for the child.^11^

Asthma is a heterogeneous disease, which can be broadly divided into two endotypes: T2 high (eosinophilic), which responds to corticosteroids and T2 low, which responds poorly to corticosteroids. Current recommendations for asthma management during pregnancy are a stepwise pharmacotherapy approach using inhaled corticosteroids (ICS).^12^ When symptoms are uncontrolled, treatment is stepped up. In the 50% of pregnant women with T2 low asthma, ICS treatment demonstrates limited efficacy,^13^ possibly leading to ICS overtreatment, which is undesirable in pregnancy. A better alternative to the ‘one size fits all’ approach to asthma management during pregnancy is required and a more personalised medicine approach is desirable. The treatable traits approach aims to identify and manage pulmonary, extrapulmonary and behavioural traits that are clinically relevant (linked to exacerbation risk or poor asthma control), identifiable, measurable and amenable to treatment.^14^ In people with severe asthma who are not pregnant, a treatable traits approach has been shown to improve asthma control, improve health-related quality of life and reduce primary care visits.^15^

Pregnant women with asthma have a high prevalence of treatable traits across all domains, including T2 high inflammation and airflow limitation causing symptoms,^13^ viral respiratory infections^16^ (pulmonary domain), obesity,^17^ depression/anxiety^18^ and rhinitis^19^ (extrapulmonary domain), smoking^9^ and poor self-management skills^20^,^22^ (behavioural domain). However, no studies have used a systematic multidimensional assessment over multiple timepoints during pregnancy to identify all traits impacting pregnant women with asthma. In addition, no studies have examined the prevalence of other traits, which are known to affect non-pregnant individuals with asthma, such as dysfunctional breathing,^23^ aspirin exacerbated respiratory disease^24^ (pulmonary domain), gastro-oesophageal reflux disease (GORD),^25 26^ obstructive sleep apnoea (OSA),^27 28^ vocal cord dysfunction (VCD),^29^ iron deficiency^30^ (extrapulmonary domain), physical inactivity,^31^ poor dietary intake^32 33^ and poor health literacy^34^ (behavioural domain), in the context of asthma and pregnancy. The link between potential treatable traits which are more prevalent in pregnant women with asthma compared with those without asthma, such as vitamin D insufficiency,^35^ hyperemesis gravidarum (HG),^36^ gestational diabetes (GD)^20^ and hypertensive disorders of pregnancy^4 6 20 35^ and risk of asthma exacerbation or poor asthma control, has not been established. It is also important to consider all traits within individual pregnant women, as some traits may correlate with others; for example, smoking is associated with an increased odds of ICS non-adherence.^37^

This study will provide insight into the prevalence of all treatable traits in pregnancy, and their association with exacerbation risk, poor asthma control and asthma-related quality of life. Filling these knowledge gaps will inform the feasibility of a treatable trait-based precision medicine approach for asthma management in the antenatal care setting and support the development of a more refined treatable trait-focused management strategy. Interventions informed by this study have the potential to improve asthma outcomes during pregnancy as well as longer term health outcomes for offspring.

## Methods and analysis

### Study design

The Treatable Traits for Asthma Management in Pregnancy (TTAP) study is a prospective, longitudinal observational cohort study.

### Population

Pregnant women with asthma attending antenatal care in a public or private hospital setting from 12 weeks gestation, in 10 different regional and metropolitan sites in Australia. The primary study site is based at the John Hunter Hospital in Newcastle, NSW. Other regional sites include Maitland, Gosford/Wyong, Queanbeyan and Wollongong hospitals. Metropolitan sites include the Royal Hospital for Women Randwick, Royal North Shore Hospital Sydney, Nepean Hospital Sydney, Royal Women’s Hospital Melbourne and Sydney Adventist Hospital (SAH). Sites were selected to ensure the cohort reflects the real-world population of women accessing care across Australia in regional and metropolitan regions, enhancing the applicability of findings across health systems and care settings, supporting translation into broader clinical practice. Recruitment quotas were set for each site based on population and birth rates.

### Inclusion criteria

The TTAP study includes women 18 years of age or older between 12 and <17 weeks’ gestation with a self-reported doctor’s diagnosis of asthma of any severity (mild, moderate, severe) who have had asthma symptoms (wheeze, cough, breathlessness) and/or taken asthma medication in the last 12 months. The gestational age of 12–<17 weeks was chosen as this corresponds to the time when women in Australia typically present for their first antenatal visit.

### Exclusion criteria

Exclusion criteria for the TTAP study include: (1) inability to attend three study visits; (2) a medical practitioner’s diagnosis of another long-term respiratory disease other than asthma (eg, cystic fibrosis, interstitial lung disease, chronic obstructive pulmonary disease); (3) participation in the study during a previous pregnancy; (4) current participation in a clinical intervention study; (5) significant life limiting comorbidity (conditions that carry a high risk of morbidity or mortality, eg, cancer, severe heart disease, severe kidney disease, advanced liver disease) (6) surrogate pregnancy; (7) cognitive impairment preventing completion of data collection forms, providing informed consent or ability to understand written and/or verbal instructions; and (8) inability to read English only in the unlikely circumstances where hospital-provided interpreter services are unable to be utilised (public hospitals in Australia provide professional interpreter services across a wide range of languages, professional interpreters can be similarly arranged through external providers at SAH).

### Recruitment

Recruitment will be conducted by the research team using site-specific recruitment processes between December 2024 and June 2027.

For sites with an existing referral pathway for maternal asthma care, an opt-in approach will be used. Midwives will be encouraged to mention the study to any women with asthma. If a woman expresses interest, midwives or clinic administrative staff would provide a study flyer with QR code and/or share the woman’s contact details with the research team for follow-up.

For sites without an established referral pathway, an opt-out approach will be implemented. The study flyer with a QR code will be included in information sent to all women booking into the hospital antenatal clinic. Women who do not wish to be contacted can opt out via email or text message. Site research staff or clinic administrative staff will then identify women with asthma, and site research staff will then contact those who have not opted out.

Additionally, study flyers with a QR code may be displayed at participating study sites, local primary care centres (i.e. general practitioners), pharmacies, ultrasound practices and health organisations. Women with asthma can choose to provide their contact information or contact the research team directly.

These diverse recruitment pathways allow for inclusion of participants of all disease severity, socioeconomic status and antenatal model of care.

### Consent

Potential participants, identified via any of the recruitment avenues, will be contacted by a member of the research team by phone. If the person agrees, a link to the participant information statement and consent form will be emailed to them. A follow-up phone call will be arranged as an opportunity to answer any further questions about the study. Participants will provide electronic consent using the REDCap (Research Electronic Data Capture) eConsent framework.^38^ Completed online consent forms will be counter-signed by the recruiting researcher and a copy provided to the participant for their records.

In addition to the main study consent, separate consent for the use of genetic material and administrative data linkage will be sought at the first study visit. Participants will be provided with these participant information statements prior to the visit to allow them time to read and consider these aspects of the study. During the visit, research staff will further explain what is involved and answer any questions. Participants will provide electronic consent using REDCap, which will then be counter-signed by research staff.

### Data collection timepoints

**Figure 1:**
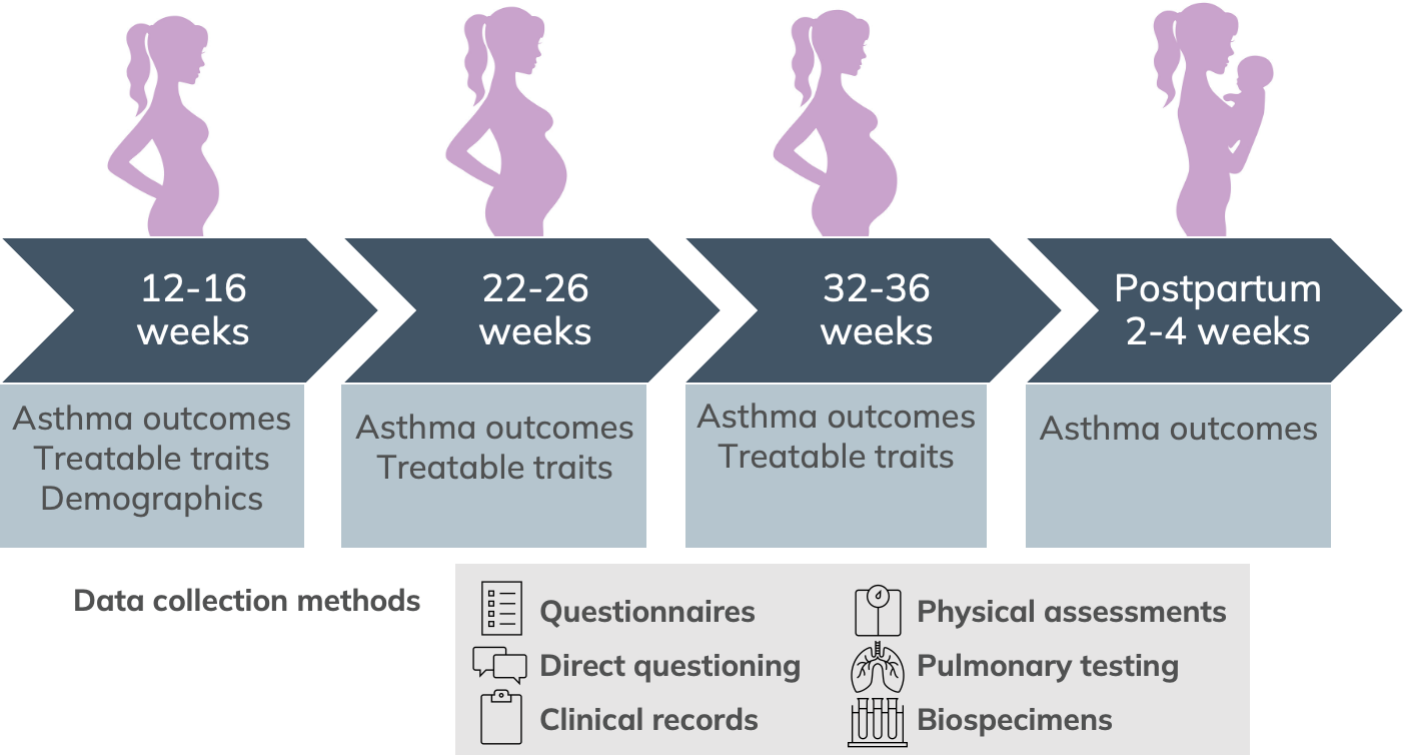
TTAP study visit schedule and data collection methods. TTAP, Treatable Traits for Asthma Management in Pregnancy.

Women will be seen at three gestational time points in their pregnancy (figure 1): 12–16 weeks, 22–26 weeks and 32–36 weeks of pregnancy. They will also be contacted by phone 2–4 weeks postpartum.

### Data collection

At each pregnancy timepoint data will be collected on the treatable traits and outcomes. Additionally, outcomes will be assessed at 2–4 weeks postpartum for the period from the last visit until birth. Treatable traits being assessed over the three domains are listed in table 1. Outcomes being assessed include asthma exacerbations (primary outcome), asthma symptom control and asthma-related quality of life (table 2). The full data collection tables are included in the supplementary material (online supplemental tables S1–S8).

**Table 1:** Trait identification markers

Trait	Trait identification marker (TIM)	V1	V2	V3	PP
Pulmonary domain					
T2 inflammation	Blood eosinophils≥260 cells/µL, or	P	P	P	
	Serum total IgE≥100 kU/L^74^, or FeNO>29 ppb^75^	P	P	P	
Airflow limitation	Spirometry FEV1<80% predicted, or FEV_1_/FVC<70%^76^	P	P	P	
Respiratory tract infections	Self-reported infections during pregnancy	A	A	A	
Aspirin exacerbated respiratory disease	Self-reported history	A			
Dysfunctional breathing	Nijmegen questionnaire score≥23^76 77^	A	A	A	
Dyspnoea	Dyspnoea-12 score: 1–10 mild, 11–20 moderate, 21–36 severe^47^	A	A	A	
Extrapulmonary domain					
Obesity/excess gestational weight gain	BMI>30 kg/m^2^ GWG above recommendations for BMI category^74 76^	P	P	P	
Depression/anxiety/stress	Self-reported history or DASS-21: Depression score>14, Anxiety score>10, Stress score>19^78^ or EPDS score 10–12 medium risk, 13–30 high risk^79^ or Heart Rate Variability (HRV) below normative range for age and gender.	A	A	A	
Rhinitis	Self-reported history, or SNOT-22 score 8–20 mild, 21–50 moderate, >50 severe^43^	A	A	A	
Gastro-oesophageal reflux disease	Self-reported symptoms or RDQ score>3^42^	A	A	A	
Obstructive sleep apnoea	Self-reported history or STOP-BANG score≥3^49^	A	A	A	
Vocal cord dysfunction	Self-reported history, or Pittsburgh VCD Index≥4^55^, or VCDQ score>12^45^	A	A	A	
Vitamin D insufficiency	Serum 25-OH vitamin D3<75 nmol	P	P	P	
Iron deficiency	Serum ferritin<30 ng/mL	P	P	P	
Anaemia	Haemoglobin<110 g/L	P	P	P	
Hyperemesis gravidarum	PUQE-24 score 7–12 mild, 13–15 severe^44^ or Self-reported and confirmed from electronic medical records	A	A	A	
Gestational hypertension	Systolic BP>140 mm Hg or Diastolic BP>90 mm Hg after 20 weeks		A	A	A
Preeclampsia	Systolic BP>140 mm Hg or Diastolic BP>90 mm Hg after 20 weeks and signs of organ involvement (from electronic medical records)		A	A	A
Gestational diabetes	75 OGTT plasma glucose (in mmol/L): fasting 5.1–6, 1 hour≥10 or 2 hour 8.5–11.0 (from electronic medical records)	A	A	A	A
Behavioural domain					
Poor asthma self-management	Inadequate inhaler technique assessed using criteria for optimal technique, or Does not possess a WAAP, or Poor asthma knowledge	A	A	A	
ICS non-adherence	TAI score≤45 poor, 46–49 intermediate^50^, or <80% prescribed doses used (digital inhaler and/or self-reported)	A	A	A	
Smoking/vaping	Self-reported, or urinary cotinine>50 ng/mL, or ECO>10 ppm (tobacco smoking only)	A	A	A	
Physical inactivity/sedentary behaviour	Physical inactivity: IPAQ-SF or accelerometry<150 MVPA min/week (<600 MET-min/week)^41^ Sedentary behaviour: ≥8 hours/day during waking hours at<100 counts/minute (accelerometry), or ≥8 hours/day sitting (IPAQ-SF)^80^	A	A	A	
Poor dietary intake	AES Australian recommended food score below median^81^			A	
Poor health literacy	SBSQ score≤5^39^	A			

This table outlines the trait identification markers, schedule and type of data collection.

Antenatal visits: V1 12-–<17 weeks, V2 22–26 weeks, V3 32–36 weeks.

Postpartum (PP) follow-up conducted via phone call at 2–4 weeks postpartum. A: Data captured at all visits (physical and telehealth) P: Data captured at in-person visits only (not telehealth).

Variable types: Binary (presence of trait based on TIM), continuous (scores).

AES, Australian Eating Survey; BMI, body mass index; BP, blood pressure; DASS-21, 21-item Depression, Anxiety and Stress Scale; ECO, exhaled carbon monoxide; EPDS, Edinburgh Postnatal/Antenatal Depression Scale; FeNO, fractional exhaled nitric oxide; FEV1, forced expiratory volume in 1 second; FVC, forced vital capacity; GWG, gestational weight gain; IgE, immunoglobulin E; IPAQ-SF, International Physical Activity Questionnaire–Short Form; MET, metabolic equivalent task; MVPA, moderate-vigorous physical activity; OGTT, Oral Glucose Tolerance Test; PUQE-24, Pregnancy-Unique Quantification of Emesis (24-hour version); RDQ, Reflux Disease Questionnaire; SBSQ, Single Item Brief Screening Question; SNOT-22, 22-item Sinonasal Outcome Test; STOP-BANG, Snoring, Tiredness, Observed Apnea, Pressure, BMI, Age, Neck circumference, Gender; TAI, Test of Adherence to Inhalers; VCD, vocal cord dysfunction; VCDQ, Vocal Cord Dysfunction Questionnaire; WAAP, Written Asthma Action Plan.

**Table 2:** Asthma outcomes

Outcome	Identification	Variable type	V1	V2	V3	PP
Exacerbations (primary outcome)	Any of the following events related to a worsening of asthma symptoms: An unplanned visit to a GP, The use of OCS medication, Admission to hospital, or Presentation to ED	Binary (any) Count (number)	A	A	A	A
Poor asthma control	ACQ-6 ≥1.5^51^p-ACT≤19^40^	Binary (uncontrolled) Continuous (scores)	A	A	A	A
Poor asthma-related quality of life	AQLQ<4^54^	Binary (poor asthma-related quality of life) Continuous (score)	A	A	A	A

This table outlines the outcomes to be assessed, schedule and type of data collection.

Antenatal visits: V1 12-–<17 weeks, V2 22-–26 weeks, V3 32-–36 weeks.

Postpartum (PP) follow-up conducted via phone call at 2-4 weeks postpartum. A: Data captured at all visit types (physical and telehealth) P: Data captured at in-person visits only (not telehealth).

ACQ-6, Asthma Control Questionnaire (6-item version); AQLQ, Asthma Quality of Life Questionnaire; ED, emergency department; GP, general practitioner; OCS, oral corticosteroids; p-ACT, Pregnancy Asthma Control Test.

Data will be collected via questionnaires, direct questioning, physical assessments, biological samples and routinely collected data from clinical records.

To minimise participant barriers, most questionnaires can be completed remotely, participants will be reimbursed for their travel and time, they will be offered flexible visit timing and visits will be aligned with routine antenatal appointments where possible. If a participant is unable to attend their scheduled visit in person during the designated gestational age window, they will be offered the option to complete the visit via telehealth. A telehealth visit will be conducted over video or phone and include direct questioning and some questionnaires; there will be no collection of biological samples and no physical assessments.

### Questionnaires

A link to online questionnaires will be emailed via REDCap to participants at recruitment (12–16 weeks), 22 and 32 weeks. Demographic information including postcode (for community level socioeconomic status), education, employment and income, housing, ethnicity, cultural identity and languages spoken will be obtained. A comprehensive health history will be included with the early survey and changes captured in subsequent surveys. Smoking, vaping and nicotine replacement therapy use will be captured at each timepoint, as will respiratory infections and vaccination status (influenza, pertussis, COVID-19, respiratory syncytial virus). Validated surveys will be used, including the Set of Brief Screening Questions (SBSQ)^39^ to assess health literacy; the Pregnancy Asthma Control Test (p-ACT)^40^ to assess asthma control; the International Physical Activity Questionnaire–Short Form (IPAQ-SF)^41^ to assess physical activity; the Reflux Disease Questionnaire (RDQ)^42^ to assess GORD; the 22-item Sinonasal Outcome Test (SNOT-22)^43^ to assess rhinitis; the Pregnancy-Unique Quantification of Emesis 24-hour version (PUQE-24)^44^ to assess HG; the Vocal Cord Dysfunction Questionnaire (VCDQ)^45^ to assess VCD; the Nijmegen Questionnaire^46^ to assess dysfunctional breathing; the Dyspnoea-12 Questionnaire^47^ to assess dyspnoea; and the Australian Eating Survey (AES)^48^ to assess poor dietary intake (visit 3 only). During study visits, participants will be asked to complete validated questionnaires in REDCap including the 21-item Depression, Anxiety and Stress Scale (DASS-21)^48^ to assess depression, anxiety and stress; the STOP-BANG^49^ to assess OSA and the Test of Adherence to Inhalers (TAI)^50^ to assess inhaler adherence (if currently using ICS). Further questionnaires will be completed on paper during study visits, including the Asthma Control Questionnaire 6-item version (ACQ-6)^51 52^ to assess asthma control; the Asthma Quality of Life Questionnaire (AQLQ)^53 54^ to assess asthma-related quality of life; and the Pittsburgh VCD Index^55^ to assess VCD (visit one only). If the visit is conducted over the phone or a video call (telehealth), the AQLQ will be posted to the participant; the ACQ-6, STOP-BANG and Pittsburgh VCD Index will be administered verbally during the telehealth visit.

### Direct questioning

Participants will be asked about their asthma history including exacerbations in the previous year, year of diagnosis and triggers at their first study visit. At each subsequent visit, participants will be asked about exacerbations since their last visit. Exacerbations will be defined as a worsening of asthma symptoms requiring additional medical intervention, indicated by the following events: (1) an unplanned visit to a general practitioner, (2) use of oral corticosteroids, (3) hospital admission or (4) presentation to an emergency department (ED). Participants will be asked about medication use for asthma including self-reported adherence at each visit. Self-management skills including inhaler technique,^56^ medication knowledge and possession of a written asthma action plan will be assessed at each study visit by a respiratory nurse or trained member of the research team as previously described^21^ and brief education provided.

Participants will be asked about diagnosed comorbidities, medications and supplement use at each study visit.

### Biological samples

All samples will be collected by a trained member of the research team, except for the stool swab, which will be collected by the participant at home. Participants will be provided with a 4N6 FLOQSwabs (Copan, Brescia, Italy) and instructions for stool collection at the second visit and asked to collect and return by mail after 1 week; this will be used to assess gut microbiome.

An ORAcollect OCR-100 (DNA Genotek Inc, Ottawa, ON, Canada) buccal swab will be collected at the first study visit for profiling of inflammation through gene expression.

Venous blood will be collected and sent to pathology (NSW Health Pathology, Douglas Hanly Moir (SAH) or the Royal Children’s Hospital Pathology in Victoria) for assessment of biomarkers including a full blood count, ferritin, 25-OH vitamin D and total IgE analysis. Further iron studies and C reactive protein will be assessed at select sites. Serum collected at the primary site will also be stored at −80°C for future assessment of biomarkers. Whole blood will be collected in PAXgene Blood RNA Tubes (PreAnalytiX GmbH, Hombrechtikon, Switzerland) at some sites for profiling of inflammation through gene expression. PAXgene tubes will be kept upright at ambient temperature for a minimum of 2 hours and maximum of 72 hours, then frozen upright at −80°C.

Hair will be collected at the second and third study visits at select sites for cortisol analysis. A small amount will be snipped from the participant’s head and stored as per the Stratech Scientific protocol.^57^ Tape will be used in place of string during collection.

Urine will be self-collected at each study visit. Urinary cotinine will be measured using IDTC-Nicotine Test Cards (CLIAwaived, Charleston, South Carolina) as an indicator of smoking status, and urine will be stored at −80 °C for future assessment of nutritional and inflammatory biomarkers at the primary site only.

All samples requiring storage will be collected only at sites where these facilities are available.

### Physical assessments

Respiratory measures, physical activity, weight and body composition will be assessed at each study visit. Height will be measured at the first study visit.

Fractional Exhaled Nitric Oxide will be measured using the NIOX VERO (Circassia AB, Uppsala, Sweden) to assess T2 airway inflammation. Spirometry will be measured using the EasyOne Air Spirometer (ndd Medical Technologies, Zurich, Switzerland) to assess airflow limitation. Exhaled carbon monoxide will be measured using the piCO Smokerlyzer Breath CO monitor (Bedfont Scientific, Maidstone, UK) to assess cigarette smoke exposure.

Maternal weight will be measured at all sites, while body composition will also be measured at the primary site using the BOD POD (COSMED, Rome, Italy), which utilises non-invasive air displacement plethysmography to estimate body fat percentage.^58^ An Actigraph WGT3X-BT (ActiGraph LLC, Pensacola, Florida) wrist accelerometer will be fitted at each study visit at three sites (John Hunter Hospital, Maitland Hospital and Gosford Hospital) and worn for 1 week to assess physical activity. Activity logbooks will be used to record activities, which may not be captured by the accelerometers. Accelerometers and logbooks will be returned via post after 1 week.

At the first visit, Hailie Smartinhaler sensors (Adherium, Auckland, New Zealand) will be fitted to salbutamol pressurised metered-dose inhaler devices and compatible turbuhaler ICS devices at four sites (John Hunter Hospital, Maitland Hospital, Gosford Hospital and Randwick Hospital) to assess asthma control and ICS adherence, respectively. These devices will be returned at the final visit.

Heart rate variability (HRV) will be measured at three sites (John Hunter Hospital, Maitland Hospital and Gosford Hospital) to assess psychological stress. During each visit, a Polar H10 (Polar Electro, Kempele, Finland) chest sensor will be used to measure HRV for 10 min in a seated position. Data will be captured and analysed using Kubios HRV Scientific Software (Kubios, Kuopio, Finland). Participants will be questioned verbally on medications, caffeine, smoking and exercise on the day of HRV measurement, to adjust for these effects.

All medical devices used in this study are listed on the Australian Register of Therapeutic Goods (ARTG) and regulated by the Therapeutic Goods Administration (TGA). ARTG details for each device used in this study are provided in the supplementary material (online supplemental table S9).

### Clinical record access

Consent to access clinical medical records will be obtained during the initial consent process.

The following data that fall within visit timeframes will be recorded: (1) routine blood pressure readings to assess gestational hypertension, (2) routine blood test results including full blood counts, iron studies and vitamin D studies.

Following birth, the following antenatal data will be extracted from the electronic medical record: (1) the Edinburgh Postnatal Depression Scale (EPDS) to assess mental health, (2) oral glucose tolerance test results to assess for GD, (3) model of antenatal care. Perinatal outcomes will also be extracted, including birth weight, congenital anomalies, preterm birth, neonatal hospitalisation and breastfeeding initiation to assess associations between traits and poor perinatal outcomes.

### Statistical analysis

Based on prior experience of recruitment in various antenatal clinic settings, we anticipate total recruitment of 650 pregnant women with asthma over 2 years. This will enable estimation of trait prevalence with high precision. Achievable precision was estimated using the standard normal approximation to the binomial distribution, a widely used approach for sample size and CI calculations for proportions. Under this method, the maximum half-width of the 95% CI is expected to range from 0.023 to 0.038 for prevalences between 10% and 90%.^59^

Prevalence and severity of traits will be reported using the trait identification marker cut points shown in table 1. Asthma outcomes will be reported as outlined in table 2.

Socioeconomic status will be assessed at community and individual levels. Area-level disadvantage will be measured using the Australian Bureau of Statistics Socio-Economic Indexes for Areas (SEIFA) Index of Relative Socio-economic Disadvantage (IRSD) quintiles derived from participants’ residential postcode at recruitment.^60^ Individual-level socioeconomic characteristics, including educational attainment, employment status, household income and housing (collected via questionnaires), will also be used.

The prevalence of treatable traits at each pregnancy timepoint will be estimated using descriptive statistics with prespecified and validated cut-offs (table 1) for continuous measures and presence of trait for binary measures. To examine associations between individual traits and asthma outcomes (table 2), generalised linear models (GLMs) will be used, with model choice dependent on outcome variable type. Secondary multivariate regressions will be conducted for each trait and outcome to adjust for confounders. Directed acyclic graphs (DAGs) will be used to identify potential confounders and minimal adjustment sets for each exposure and outcome model.^61^ The main confounding set will include demographic variables (socioeconomic status at community and individual levels, maternal age, remoteness, body mass index (BMI)) and pregnancy-related characteristics, including antenatal model of care, gestational weight gain, relevant environmental exposures, smoking during pregnancy (except in models where smoking is the trait of interest) and other treatable traits as relevant. An example DAG is found in the supplement (online supplemental figure S1).

Bayesian model averaging (BMA)^62^ will be used to address uncertainty in the selection of correlated treatable traits, while adjustment for confounders will be determined a priori using DAGs and fixed across all models. BMA will be implemented using model forms as appropriate for outcome.^29^ This approach provides posterior inclusion probabilities for each trait, offering a probabilistic summary of their relative importance across competing models.

For traits measured at multiple time points and for which differences are observed, univariate group-based trajectory modelling (GBTM) will be used for each trait to examine the course (or trajectory) of the trait throughout the pregnancy and identify individual trait trajectory subgroups.^63^,^65^ Univariate GBTM will also be applied to the outcomes of asthma control and asthma-related quality of life. Trajectory group membership will then be used as a categorical variable for GLM.

For each trait, separate regression models will be fitted to examine the relationship between trajectory memberships with outcomes, with model choice dependent on outcome variable type (table 2) or trajectory group membership (categorical) for the outcomes of asthma control and asthma-related quality of life.

Associations between traits and adverse perinatal outcomes will be examined using linear or logistic regression modelling as appropriate, adjusting for relevant covariates.

All GLMs will be determined by variable type; logistic regression will be used for all binary and categorical variables; negative binomial regression will be used for count variables; and linear regression will be used for continuous variables.^66^,^68^

Prespecified subgroup analyses will be conducted to explore potential effect modification by baseline ICS use, remoteness (regional vs metropolitan sites), BMI category and antenatal model of care. These subgroup analyses are hypothesis-driven and will be reported irrespective of statistical significance. Effect modification will be assessed using interaction terms included in multivariable models. Interaction p values will be interpreted cautiously, with emphasis on effect sizes and CIs, recognising that interaction tests may be underpowered.

Sensitivity analyses will be conducted to assess the robustness of study findings. As the primary study site in Newcastle collects more detailed assessments of selected traits in addition to core measurements obtained at all sites, sensitivity analyses will be restricted to Newcastle participants. This will allow assessment of potential measurement bias by comparing results derived from core measurements across all sites with those based on the more detailed assessments in Newcastle.

Missing data will be handled using complete case methods for the primary analyses, given the low anticipated level of attrition (10%–15%). Sensitivity analyses will be conducted using multiple imputation under a missing at random assumption for key outcomes and analyses. Imputed datasets will be generated using chained equations incorporating all variables included in the analysis models.

All statistical tests will be two-sided, with a nominal significance level of 0.05. Results will be presented as point estimates with 95% CIs, with p values reported where relevant but interpreted cautiously. Given the large number of traits examined, emphasis will be placed on the magnitude, precision and consistency of effect estimates across models and time points rather than statistical significance alone. No formal adjustment for multiple comparisons is planned, and findings from secondary and subgroup analyses will be interpreted as hypothesis-generating.

To assess external validity, cohort representativeness will be assessed by comparing key maternal demographic and antenatal characteristics with publicly available national and state perinatal data from the Australian Institute of Health and Welfare and Perinatal Data Collections from NSW and Victoria.^69^,^71^

This protocol is reported in accordance with the Strengthening the Reporting of Observational Studies in Epidemiology (STROBE) guidelines. A completed STROBE checklist is provided in the supplementary material (online supplemental table S10).

## Ethics and dissemination

### Ethics and governance

The TTAP study was approved by the Hunter New England Human Research Ethics Committee on 30 July 2024 (Reference: 2024/ETH01289) and registered with The University of Newcastle (Reference: R-2024-0071). Site-Specific Approvals were obtained for all participating hospitals, including John Hunter Hospital, Newcastle (2024/STE 02376); Royal North Shore Hospital, Sydney (2024/STE 02378); Royal Hospital for Women, Randwick (2024/STE 02380); Sydney Adventist Hospital (Project Code: 2025–003); Royal Women’s Hospital, Melbourne (SSA/113217/RWHV-2025-474363); Royal Melbourne Hospital, Melbourne (SSA/113217/MH-2025-467107); Gosford Hospital and Wyong Hospital (2024/STE 03310); Maitland Hospital (2024/STE 02377); Wollongong Hospital (2024/STE 02383); Queanbeyan Hospital (2024/STE 02381); and Nepean Hospital, Penrith (2024/STE 02382). All participants provided written informed consent prior to enrolment, and the study was conducted in accordance with the principles of the Declaration of Helsinki and relevant national guidelines.

### Quality control and participant safety

All TTAP study staff will be trained and required to demonstrate sufficient ability in data collection, biological sample collection and informed consent processes, and a detailed study manual will be provided for reference. Staff will be required to attend a venepuncture training course approved by their Local Health District. Pulmonary function testing, asthma self-management and asthma education training will be provided by an experienced respiratory nurse (KS). Any findings which may be clinically relevant will be reviewed by clinical staff and referred to site antenatal healthcare providers and/or a respiratory specialist if necessary.

### Participant compensation/reimbursement

Participants are reimbursed for their time and travel expenses, as approved by the Human Research Ethics Committee.

### Data deposition and curation

TTAP study data will be collected and managed using REDCap, a secure, web-based platform for research data capture, hosted at The Hunter Medical Research Institute (HMRI).^72 73^ All participants will be assigned a unique, reidentifiable Study ID code. During the study period, participant data will be identifiable by study staff at each participating site, the database custodian and the study manager. Following study completion, only the database custodian will be able to reidentify data. The database custodian will not directly analyse data. Data will be stored for a minimum of 15 years. Following completion of the project, an indexed and retrievable dataset will be downloaded from REDcap and stored securely.

### Study governance

A Steering Committee, consisting of the lead Chief Investigator, Study Manager and Principal Investigators from each site will be responsible for implementing the study protocol and ensuring the safety and quality of the study. The Steering Committee is responsible for monitoring study objectives and outcomes and ensuring the study is conducted according to International Conference on Harmonisation Good Clinical Practice. Steering Group meetings will be held bi-monthly during the early recruitment phase and quarterly thereafter.

Requests for data use will be reviewed and approved by the Steering Committee in accordance with the study’s data access policy.

### Publication and dissemination

Once sufficient data have been collected, outcomes will be published in peer-reviewed journals with the use of an open licence and presented at scientific conferences and other professional forums. Findings will also be disseminated online via the Asthma in Pregnancy Toolkit website (https://asthmapregnancytoolkit.org.au/).

At the conclusion of the study and publication of manuscripts, all participants will be emailed a summary of the main study findings and links to the manuscripts produced.

## Supplementary material

10.1136/bmjopen-2025-115626 online supplemental file 1
