# Supplementary material for: Treatable Traits for Asthma Management in Pregnancy (TTAP): protocol for an Australian multicentre prospective observational cohort study
Source: BMJ Open. 2026 Jun 2;16(6):e115626. doi: 10.1136/bmjopen-2025-115626 (PMC13239357; doi:10.1136/bmjopen-2025-115626)
Supplement: online supplemental file 1 [file bmjopen-16-6-s001.docx]

# Supplementary Material

# TTAP Study Data Collection Tables (Tables S1-S8)

Treatable Traits for Asthma Management in Pregnancy (TTAP) – Data collection tables aligned with protocol. Concomitant medication use is recorded at each visit via direct questioning.

## Table S1. Participant Characteristics and Demographics

| Variable | Instrument / Source | Timing |
| --- | --- | --- |
| Maternal age | Questionnaire | V1 |
| Education, employment, income | Questionnaire | V1 |
| Housing, remoteness | Questionnaire | V1 |
| Ethnicity, cultural identity | Questionnaire | V1 |
| Language spoken | Questionnaire | V1 |
| SEIFA IRSD quintile | Derived from postal code at recruitment | Enrolment |
| Comorbidities | Questionnaire | V1-V3 |

## Table S2. Asthma History, Management, and Concomitant Medications

| Variable | Instrument / Source | Timing |
| --- | --- | --- |
| Asthma diagnosis | Self-report | V1 |
| Year of diagnosis | Direct questioning | V1 |
| Asthma triggers | Direct questioning | V1 |
| Asthma medication use | Direct questioning | V1–PP |
| Concomitant medication use | Direct questioning | V1–V3 |

## Table S3. Asthma Outcomes

| Variable | Instrument / Source | Timing |
| --- | --- | --- |
| Asthma exacerbations | Self-report of GP visit, OCS, ED or hospitalisation | V1–PP |
| Asthma control | ACQ-6, p-ACT | V1–PP |
| Asthma-related quality of life | AQLQ | V1–PP |

## Table S4. Pulmonary Treatable Traits

| Variable | Instrument / Source | Timing |
| --- | --- | --- |
| T2 inflammation | Blood eosinophils, serum IgE, FeNO | V1–V3 |
| Airflow limitation | Spirometry (FEV1, FEV1/FVC) | V1–V3 |
| Respiratory infections | Questionnaire | V1–V3 |
| Aspirin exacerbated respiratory disease | Direct questioning | V1 |
| Dysfunctional breathing | Nijmegen Questionnaire | V1–V3 |
| Dyspnoea | Dyspnoea-12 | V1–V3 |

## Table S5. Extra-pulmonary Treatable Traits

| Variable | Instrument / Source | Timing |
| --- | --- | --- |
| Obesity / gestational weight gain | Height, weight, BMI | V1–V3 |
| Depression, anxiety, stress | DASS-21, EPDS, HRV | V1–PP |
| Rhinitis | SNOT-22 | V1–V3 |
| Gastroesophageal reflux disease | RDQ | V1–V3 |
| Obstructive sleep apnoea | STOP-BANG | V1–V3 |
| Vocal cord dysfunction | VCDQ, Pittsburgh VCD Index | V1–V3 |
| Vitamin D insufficiency | Serum 25-OH Vitamin D3 | V1-V3 |
| Iron deficiency | Serum ferritin | V1-V3 |
| Anaemia | Venous hemoglobin | V1-V3 |
| Hyperemesis gravidarum | PUQE-24 | V1-V3 |
| Gestational hypertension | Electronic medical records | V2-PP |
| Preeclampsia | Electronic medical records | V2-PP |
| Gestational diabetes | Electronic medical records | V1-PP |

## Table S6. Behavioural Treatable Traits

| Variable | Instrument / Source | Timing |
| --- | --- | --- |
| Poor asthma self-management | Inhaler technique, knowledge, WAAP | V1–V3 |
| ICS non-adherence | TAI, digital inhaler sensor | V1–V3 |
| Smoking / vaping | Self-report, urinary cotinine, eCO | V1–V3 |
| Physical activity / sedentary behaviour | IPAQ-SF, accelerometry | V1–V3 |
| Poor dietary intake | Australian Eating Survey | V3 |
| Poor health literacy | SBSQ | V1 |

## Table S7. Biological Samples

| **Variable** | **Instrument / Source** | **Timing** |
| --- | --- | --- |
| Venous blood | Biomarker assessment | V1–V3 |
| Buccal swab | Gene expression profiling | V1 |
| Stool | Gut microbiome | V2 |
| Urine | Cotinine and stored biomarkers | V1–V3 |
| Hair | Cortisol | V2–V3 |

## Table S8. Clinical Records and Perinatal Outcomes

| **Variable** | **Instrument / Source** | **Timing** |
| --- | --- | --- |
| Blood pressure measurements | Electronic medical record | V1-V3 |
| Routine antenatal blood tests | Electronic medical record | V1-V3 |
| Antenatal model of care | Electronic medical record | PP |
| Birth weight | Electronic medical record | PP |
| Congenital anomalies | Electronic medical record | PP |
| Preterm birth | Electronic medical record | PP |
| Neonatal hospitalisation | Electronic medical record | PP |
| Breastfeeding initiation | Electronic medical record | PP |

Footnotes (applies to Tables S1–S8):

**Visit timepoints:** V1 = 12–<17 weeks’ gestation (baseline antenatal visit); V2 = 22–26 weeks’ gestation (mid‑pregnancy visit); V3 = 32–36 weeks’ gestation (late‑pregnancy visit); PP = 2–4 weeks postpartum (telephone follow‑up).

**Abbreviations:** SEIFA, Socio‑Economic Indexes for Areas (developed by the Australian Bureau of Statistics using census data); IRSD, Index of Relative Socioeconomic Disadvantage; ACQ‑6, Asthma Control Questionnaire (6‑item); AQLQ, Asthma Quality of Life Questionnaire; BMI, body mass index; DASS‑21, Depression Anxiety Stress Scale (21‑item); eCO, exhaled carbon monoxide; ED, emergency department; EPDS, Edinburgh Postnatal Depression Scale; FeNO, fractional exhaled nitric oxide; FEV1, forced expiratory volume in 1 second; FVC, forced vital capacity; GP, general practitioner; HRV, heart rate variability; ICS, inhaled corticosteroids; IPAQ‑SF, International Physical Activity Questionnaire–Short Form; OCS, oral corticosteroids; p‑ACT, Pregnancy Asthma Control Test; RDQ, Reflux Disease Questionnaire; SBSQ, Single‑item Brief Screening Question; SNOT‑22, 22‑item Sinonasal Outcome Test; STOP‑BANG, Snoring, Tiredness, Observed apnoea, Pressure, BMI, Age, Neck circumference and Gender questionnaire; TAI, Test of Adherence to Inhalers; VCDQ, Vocal Cord Dysfunction Questionnaire; WAAP, Written Asthma Action Plan.

# Table S9. Medical Devices used in the TTAP Study and Regulatory Status

| **Device Name** | **Manufacturer Name** | **Device Type** | **Intended Use in Study** | **ARTG Status** |
| --- | --- | --- | --- | --- |
| NIOX VERO | NIOX AB | FeNO analyser | Measurement of FeNO to assess Type-2 inflammation | **Listed:** Class I  Instrument/analyser IVDs  **ID:** 197655  **Sponsor:** Inmunotek Australia Pty Ltd |
| EasyOne Air Spirometer | NDD Medizintechnik AG | Diagnostic spirometer | Spirometry measurements | **Listed:** Class IIa  EasyOne Pro – Pulmonary function analyser, adult  **ID:** 131960  **Sponsor:** Innervate Pty Ltd |
| piCO (Smokerlyzer) | Bedfont Scientific Ltd | CO breath analyser | Measurement of exhaled CO for smoking status assessment | **Listed:** Class IIa  **ID:** 178801  Analyser, gas, carbon monoxide  **Sponsor:** Innervate Pty Ltd |
| COSMED BODPOD | Akern Srl | Body composition analyser (air displacement plethysomography) | Assessment of body composition | **Listed:** Class IIa  **ID:** 383368  Body fat/lean ratio analyser  **Sponsor:** Cosmed Asia-Pacific |
| Actigraph wGT3X-BT | Actigraph LLC | Activity monitor / wearable sensor | Passive measurement of physical activity | **Listed:** Class I  **ID:** 320859  Patient data recorder, long-term, physical activity  **Sponsor:** Emergo Asia Pacific Pty Ltd |
| Hailie Smartinhaler | Adherium NZ Ltd | Inhaler monitoring device | Recording inhaler use for adherence assessment | **Listed:** Class I  **ID:** 402070  Inhaler dose sensor  **Sponsor:** Adherium Ltd |
| Polar H10 chest sensor | Polar Electro Oy | Heart rate sensor | Non-diagnostic passive physiological monitoring of HRV | Not listed |

Footnotes (Applies to Table S9.)

**Abbreviations and their definitions:** IVD = In Vitro Diagnostic, ARTG = Australian Register of Therapeutic Goods, FeNO = Fractional Exhaled Nitric Oxide, CO= Carbon Monoxide, HRV = Heart Rate Variability

# Table S10. STROBE checklist

Study: Treatable Traits for Asthma Management in Pregnancy (TTAP) protocol

| **Item No** | **STROBE item** | **Recommendation** | **Location in manuscript** |
| --- | --- | --- | --- |
| 1a | Title/Abstract | Study design indicated in title/abstract | Title; Abstract – Methods (p.2) |
| 1b | Title/Abstract | Informative summary | Abstract (p.2) |
| 2 | Background | Scientific background and rationale | Introduction (pp.4-5) |
| 3 | Objectives | Specific objectives/hypotheses | Introduction (final paragraph p.5) |
| 4 | Study design | Key elements presented early | Methods – Study design (p.6) |
| 5 | Setting | Setting, locations, dates | Methods – Population; Recruitment (pp.6-7) |
| 6a | Participants | Eligibility and selection methods | Methods – Inclusion/Exclusion; Recruitment (pp.6-7) |
| 6b | Participants | Matching criteria | Not applicable |
| 7 | Variables | Outcomes, exposures, confounders defined | Methods – Data collection; Statistical analysis; Tables 1–2 (pp.8-15) |
| 8 | Data sources | Sources and measurement methods | Methods – Data collection; Tables 1-2 (pp.8-15) |
| 9 | Bias | Efforts to address bias | Strengths & limitations; Statistical analysis (pp.3, 15-17) |
| 10 | Study size | Sample size rationale | Statistical analysis (p.13) |
| 11 | Quantitative variables | Handling of quantitative variables | Statistical analysis; Tables 1–2 (pp.13-17) |
| 12a | Statistical methods | Overall statistical methods | Statistical analysis (pp.13-17) |
| 12b | Statistical methods | Subgroups & interactions | Statistical analysis (pp.13-17) |
| 12c | Statistical methods | Missing data | Statistical analysis (p.17) |
| 12d | Statistical methods | Loss to follow-up | Statistical analysis (pp.13-17) |
| 12e | Statistical methods | Sensitivity analyses | Statistical analysis (pp.13-17) |
| 13–17 | Results | Results reporting | Planned future manuscript |
| 18–21 | Discussion | Interpretation & generalisability | Planned future manuscript |
| 22 | Funding | Funding source and role | Funding statement (p.20) |

# Figure S1: Directed Acyclic Graph for Gastroesophageal Reflux Disease


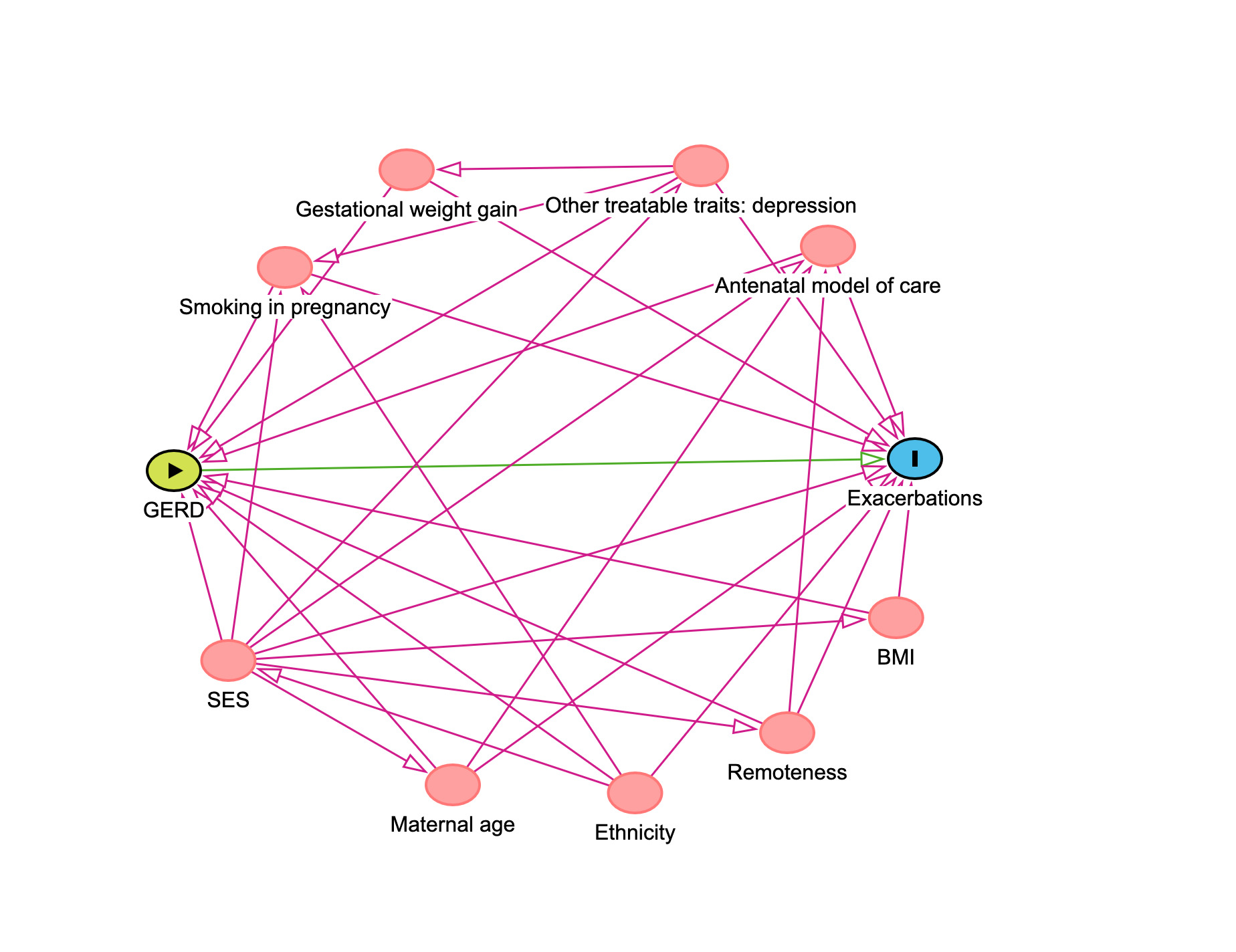


Figure S1. Example directed acyclic graph for

**Abbreviations:** GERD = gastroesophageal reflux disease, SES = Socioeconomic status, BMI = Body mass index
