# Supplementary material for: Treatable Traits for Asthma Management in Pregnancy (TTAP): protocol for an Australian multicentre prospective observational cohort study
Source: BMJ Open. 2026 Jun 2;16(6):e115626. doi: 10.1136/bmjopen-2025-115626 (PMC13239357; doi:10.1136/bmjopen-2025-115626)
Supplement: Supplementary file 2 [file bmjopen-2025-115626.reviewer_comments.pdf]

PEER REVIEW HISTORY

BMJ Open publishes all reviews undertaken for accepted manuscripts. Reviewers are asked to complete a checklist review form and are provided with free text boxes to elaborate on their assessment. These free text comments are reproduced below.

ARTICLE DETAILS

Title (Provisional)

Treatable Traits for Asthma Management in Pregnancy (TTAP): protocol for an Australian multicentre prospective observational cohort study

Authors

Grehan, Jane E; Bristow, Janet; Steel, Kelly; Brew, Bronwyn; Peek, Michael J; Robijn, Annelies L; Barrett, Helen L; Seeho, Sean; Fotheringham, Penelope; Atchan, Marjorie; Harvey, Soriah M; Samuel, Sameh R; McDonald, Vanessa; Jensen, Megan E.; Holliday, Elizabeth; Rees, Megan; Elvidge, Elissa; Vining, Lesley; Chapman, Kathleen; Joshi, Esha; Jones, Andrew C; Bowman, Rebekah; Brereton, Christopher J; Price, Sarah AL; McNamara, Kelly A; Beech, Amanda; Pennell, Craig; Murphy, Vanessa E

VERSION 1 - REVIEW

**Reviewer**	**1**
**Name**	**Antoniu, Sabina**
**Affiliation**	**Grigore T. Popa University of Medicine and Pharmacy**
**Date**	**01-Feb-2026**
**COI**	**None**

---

please briefly comment why you only considered gestational age of 12-17 weeks

---

**Reviewer**	**2**
**Name**	**Jordan, Sue**
**Affiliation**	**Swansea University, Nursing**
**Date**	**14-Feb-2026**
**COI**	**None**

Thank you for the opportunity to read this protocol for an observational study. The topic is important, as there is considerable opportunity to improve routine care. The manuscript could be improved by including further details and discussion of the limitations of the study.

Abstract

The abstract indicates 3 assessment points, but the flow diagram p.26 includes a post partum assessment.

Exclusion criteria

Please specify “another respiratory disease”, “high dependence”, “life-limiting co-morbidity”.

Exclusion of the most ill women is a serious limitation, which affects the value of the study, and should be discussed. These are the women who need most help. Exclusion of women using recreational medicines restricts the real-world value of the study; this limitation should be explained and acknowledged.

Will English be the only language available? If so, this should be discussed in the limitations section.

Recruitment

• Will researchers be directly involved in recruitment?

• Is consent to be taken twice? Please clarify.

• Will women be reimbursed for their time completing the questionnaires?

• Dates of study should be included here.

• Will all centres have a recruitment quota to balance the study sample?

Outcomes

• Obstetric and perinatal outcomes, including congenital anomalies, and successful breastfeeding would strengthen the study. Absence of these outcomes is an important limitation.

• Greater emphasis on the post partum visit would acknowledge the high risks at this time.

• What follow up after 2 weeks post partum is planned?

• I did not see where data on all asthma medicines were included in tables. Use of medicines exacerbating asthma should also be captured.

CE registration details should be supplied for the instruments used, perhaps as an appendix. Why will only some sites collect certain samples?

Sample size calculation

More details and a reference for the method used should be added.

Statistical analysis

What is the primary outcome?

Model covariates for each outcome should be pre-specified. The DAGs should be included in the protocol paper. Text on p.16 seems to be repeated. A bulleted list of analyses with

covariates would be clearer for readers. References to methodological precedents would strengthen the text.

How will socio-economic status be accounted? If this isn't included in data collection, it should be discussed in the limitations section.

If no subgroup and sensitivity analyses are planned, this should be stated.

How will missing data and 'loss to follow up' be accounted?

Discussion

A short description of how the associations explored in this study will improve care is needed to complete this paper.

The strengths and weaknesses of the study design should also be explored. The detailed examination of biomarkers should contribute unique insights, and is an important strength. Some of the serious limitations are mentioned in the summary, but need greater exploration. What strategies will be deployed to reduce volunteer selection bias? How will population representivity be checked? Collider bias is an important threat to volunteer cohorts. How will this be minimised? Which of the traits identified are mediators or moderators? How will this be accommodated in the analyses?

General

References might usefully be updated.

I did not find a reporting guideline. The EQUATOR website recommends the STROBE checklist for protocols for observational studies.

Is the study registered with a database?

Ethical approvals should be dated.

The responsible statistician should be named.

Language

'Long-term conditions' might be considered less pejorative than 'common chronic diseases'.

I suggest all summary bullet points are presented as sentences.

I noted grammatical errors: e.g. p.7 line31/32; p.8 line22; p.12 line 4; p.19 line 30.

Minor point

Some abbreviations were not evident in the table foot notes.

VERSION 1 - AUTHOR RESPONSE

Response to Reviewers

<u>Response to Reviewer: 1</u>

***Comment 1:* please briefly comment why you only considered gestational age of 12-17 weeks**

Response: We thank the reviewer for this question. We focused on a gestational age window of 12–17 weeks' because this represents the earliest timepoint at which women typically present for antenatal care across our study sites in Australia. While earlier intervention is clinically desirable, first-presentation varies between hospitals, and 12–17-week window captures this variability. In addition, a general population comparison cohort completed their first study visit at 14 weeks' gestation (*Grace et al*., Newcastle 1000 (NEW1000) Study: an Australian population-based prospective pregnancy cohort study design and protocol, *BMJ Open*, 2023), aligning our recruitment window as closely as possible allows more valid comparisons between cohorts, if required.

We have added this statement to the Inclusion Criteria on page 6:

"*Gestational period of 12-17 weeks was chosen for recruitment as this is when women in Australia typically present for their first antenatal visit.*"

<u>Response to Reviewer 2</u>

Comments on abstract

***Comment 1:* The manuscript could be improved by including further details and discussion of the limitations of the study.**

Response: We would like to thank the reviewer for this useful feedback. Further discussion as to how the limitations of the study will be addressed is now included in the "statistical analysis" and "recruitment" sections on pages 6, 7, 12 and 13. Please also see the answers to comments 4, 10, 19, and 23.

***Comment 2:* The abstract indicates 3 assessment points, but the flow diagram p.26 includes a post-partum assessment.**

Response: Thank you for this observation, this was an oversight on our behalf. We have now included the post-partum assessment in the "methods and analysis" section of the abstract page 2 ("*A follow-up assessment of asthma outcomes is conducted at 2-4 weeks post-partum*"), as well as the first paragraph of data collection page 8 ('*Additionally, outcomes will be assessed at 2-4 weeks postpartum*').

Comments on exclusion criteria

***Comment 3:* Please specify "another respiratory disease", "high dependence", "life-limiting co-morbidity". Exclusion of the most ill women is a serious limitation, which affects the value of the study, and should be discussed. These are the women who need most help. Exclusion of women using recreational medicines restricts the real-world value of the study; this limitation should be explained and acknowledged.**

Response: We thank the reviewer for this constructive and important comment. For the purpose of this study "another respiratory disease" refers to any long-term respiratory disease that is not asthma (eg cystic fibrosis, interstitial lung disease, chronic obstructive pulmonary disease), "life-limiting co-morbidity" refers to any medical conditions that carry a high risk of severe morbidity or mortality during pregnancy (eg. cancer, severe heart disease, severe kidney disease, advanced liver disease). This has been added to the manuscript under "Exclusion Criteria" on page 6.
In response to this review, the exclusion criteria relating to "high-dependence" and recreational medicine use were further discussed by the study steering committee and removed early in the study, pending ethics approval. Recruitment had already commenced at the time of this amendment; however, the change will be implemented early and prior to the majority of participant enrolment. This modification is expected to increase the generalisability of the cohort and is unlikely to materially impact study validity. The manuscript has been updated to reflect the current inclusion and exclusion criteria (pages 6 and 7).
We note that we are intentionally aiming to recruit women with all disease severity and receiving all treatment modality. Women with severe asthma will not be excluded from the study.

Comment 4: **Will English be the only language available? If so, this should be discussed in the limitations section.**
Response:
We thank the reviewer for this important comment. English will not be the only language available for study participation. Professional interpreter services are routinely used in Australian public hospitals and at Sydney Adventist Hospital to support communication with participants who do not speak English fluently. A woman would only be excluded on language grounds in the uncommon circumstance where professional interpreter services are unable to be utilised for consent or study procedures. This has now been clarified in the manuscript under "Exclusion Criteria" on page 7: *"inability to read English only in the unlikely circumstances where hospital-provided interpreter services are unable to be utilised (public hospitals in Australia provide professional interpreter services across a wide range of languages, professional interpreters can be similarly arranged through external providers at SAN)."*

Comments on recruitment
Comment 5: **Will researchers be directly involved in recruitment?**
Response: Researchers will be contacting, consenting and conducting study visits with participants. Clinical care will in no way impacted by this study. We have added a statement on page 7 to this effect: "*Recruitment will be conducted by the research team using site-specific recruitment processes between December 2024 and June 2027."*

Comment 6: **Is consent to be taken twice? Please clarify.**
Response: We thank the reviewer for this opportunity to clarify consent procedures. Women are consented to the main study digitally prior to their first visit. During their first visit staff will discuss linkage of their health information and use of their genetic material, these aspects of the study have separate consent forms which can be completed by the participant during this visit. This has been further clarified in the second paragraph of the "Consent" section on page 8: *"In addition to the main study consent, separate consent for the use of genetic material and administrative data linkage will be sought at the first study visit."*

Comment 7: **Will women be reimbursed for their time completing the questionnaires?**
Response: Women are reimbursed for their time and travel for each visit. A section labelled "Participant compensation/reimbursement" has been added to the ethics and dissemination section on page 19 to address this: *"Participants are reimbursed for their time and travel expenses, as approved by the Human Research Ethics Committee."*

Comment 8: **Dates of study should be included here.**
Response: We have now added the dates of the study to the first paragraph of the "Recruitment" section on page 7: *"Recruitment will be conducted by the research team using site-specific recruitment processes between December 2024 and June 2027."*

Comment 9: **Will all centres have a recruitment quota to balance the study sample?**
Response: We thank the reviewer for this constructive comment. Recruitment quotas for each site were determined based on the annual number of live births at that site which vary, therefore the quotas are different. The study was intentionally designed with a strong emphasis on regional centres, hence we included a number of regional sites with lower quotas due to smaller populations. Additionally, the sampling strategy was weighted to reflect the ethnic diversity of these regional populations. This has been further expanded on in the "Population" section page 6: *"Sites were selected to ensure the cohort reflects the real-world population of women accessing care across Australia in regional and metropolitan regions, enhancing the applicability of findings across health systems and care settings, supporting translation into broader clinical practice. Recruitment quotas were set for each site based on population and birth rates."*

Comments on outcomes

***Comment 10:* Obstetric and perinatal outcomes, including congenital anomalies, and successful breastfeeding would strengthen the study. Absence of these outcomes is an important limitation.**
Response: We thank the reviewer for this helpful suggestion. We are currently collecting information on congenital anomalies, and this was accidentally omitted from the manuscript. This has now been added to the “Clinical record access” section (page 13).
We agree that breastfeeding is an important outcome to collect, and initiation of breastfeeding will be added to perinatal outcomes for this study. An extensive postpartum follow-up sub-study which includes further breastfeeding outcomes is planned. This sub-study will have its own protocol.

***Comment 11:* Greater emphasis on the post-partum visit would acknowledge the high risks at this time.**
Response: We thank the reviewer for this useful suggestion. The purpose of the postpartum visit outlined in this manuscript is to capture asthma outcomes that occurred during the final weeks of pregnancy following the last visit rather than a focus on the postnatal period *per se*, this has been clarified in the manuscript on page 8 under “Data Collection”: *“Additionally, outcomes will be assessed at 2-4 weeks postpartum for the period from the last visit until birth.”*
We agree that the postpartum period is a high-risk time and a separate post-partum sub study with its own protocol is planned to address this.

***Comment 12:* What follow up after 2 weeks post-partum is planned?**
Response: Ethics approval has recently been granted for a 12-month postpartum follow-up study, this study will track participants through the first year after birth, assessing asthma outcomes, treatable traits, and breastfeeding outcomes.

***Comment 13:* I did not see where data on all asthma medicines were included in tables. Use of medicines exacerbating asthma should also be captured.**
Response: We thank the reviewer for identifying this. Asthma medications as well as concomitant medications and supplements are captured at each visit as specified under “Direct Questioning” on page 10. These were not included in the tables as they are not a trait or an outcome. An additional table outlining all data captured (including medications) at each visit is now included in the supplementary material (Table S2).

***Comment 14*: CE registration details should be supplied for the instruments used, perhaps as an appendix. Why will only some sites collect certain samples?**
Response: We thank the reviewer for these comments, our responses are below.

<u>CE registration details</u>

As the study is conducted in Australia, Australian regulatory details apply. In Australia, medical devices must be included in the Australian Register of Therapeutic Goods (ARTG) to be lawfully supplied, this is regulated by the Therapeutic Goods Administration (TGA). A table outlining the ARTG details of each device is now included in the supplementary material (Table S9). The following text has now been included in the "Physical assessments" section of the manuscript on page 12: *"All medical devices used in this study are listed on the Australian Register of Therapeutic Goods (ARTG) and regulated by the Therapeutic Goods Administration (TGA). ARTG details for each device used in this study are provided in the supplementary material (Table S9)."*

Site sample collection

Some samples are not collected at sites where processing and storage facilities are not available. This has now been addressed under the "Biological samples" section of the manuscript on page 11: "All samples requiring storage will be collected only at sites where these facilities are available."

Comment 15: **Sample size calculation - More details and a reference for the method used should be added.**
Response: We thank the reviewer for this important comment. We estimated the achievable precision for prevalence estimates using the standard normal approximation to the binomial distribution, a widely used method for sample size and confidence interval calculations for proportions. Under this approach, the approximate 95% confidence interval half width is given by

$$h = 1.96\sqrt{p(1-p)/n}$$

This method is described in standard statistical texts on sample-size determination (e.g., Fleiss et al., Statistical Methods for Rates and Proportions, 3rd ed., Wiley, 2003). We have now added the following to the "Statistical analysis" section page 13: "*Achievable precision was estimated using the standard normal approximation to the binomial distribution, a widely used approach for sample size and confidence interval calculations for proportions."*

Comments on Statistical analysis
Comment 16: **What is the primary outcome?**
Response: The primary outcome is Exacerbations as a binary outcome (occurred/didn't occur). This has been clarified in the manuscript under the abstract "Methods and analysis" section on page 2: *"The outcomes assessed are asthma exacerbations requiring medical intervention (primary outcome), asthma symptom control and asthma-related quality of life."* It has also been clarified in Table 2 (page 15) and addressed under the "Data collection" section on page 8: *"Outcomes being assessed include asthma exacerbations (primary outcome)"*

Comment 17: **Model covariates for each outcome should be pre-specified. The DAGs should be included in the protocol paper. Text on p.16 seems to be repeated. A bulleted list of analyses with covariates would be clearer for readers.**
Response: We thank the reviewer for these constructive suggestions. Given the large number of exposures (treatable traits) and the complex interrelationships between traits when considered as covariates for one another, it was not feasible to present a separate DAG for each trait. An illustrative example DAG is provided in the Supplementary Material (Figure S1) and a reference to this is now included in the manuscript under statistical analysis on page 16.
Thanks for the useful feedback regarding the text on page 16; description of GLM modelling for all analysis is now summarised in a single paragraph (page 16): *"All GLMs will be determined by variable type; logistic regression will be used for all binary and categorical variables; negative binomial regression will be used for count variables; and linear regression will be used for continuous variables"*. Variable type (which determines the GLM type) is included in Table 2.

Comment 18: **References to methodological precedents would strengthen the text.**
Response: We thank the reviewer for this constructive feedback. We have now added references to methodology in the "Statistical analysis" section pages 13-17.

Comment 19: **How will socio-economic status be accounted? If this isn't included in data collection, it should be discussed in the limitations section.**
Response: We will assess socioeconomic status at community and individual levels. On the community level, socioeconomic status will be assessed using area-level Socio-Economic Indexes for Areas (SEIFA) developed by the Australian Bureau of Statistics (ABS) using Census data. Participants' postal codes at recruitment will be used to derive SEIFA Index of Relative Socio-economic Disadvantage (IRSD) quintiles. According to the Australian Bureau of Statistics "IRSD is a general socio-economic index that summarises a range of information about the economic and social conditions of people and households within an area. IRSD only includes measures of relative disadvantage. A low score indicates relatively greater disadvantage. For example, an area could have a low score if there are: many households with low income, or many people without qualifications, and many people in low skilled occupations." (https://www.abs.gov.au/statistics/people/people-and-communities/socio-economic-indexes-areas-seifa-australia/latest-release).
Further information on this including a reference has now been included in the manuscript under "Questionnaires" on page 9: *"Demographic information including postcode (for community level socio-economic status)"*

Individual level socioeconomic characteristics including educational attainment, employment status, household income and housing will be captured using questionnaires as stated in paragraph 1 of the “Questionnaires” section on page 9.

The following additional information has now been added to the manuscript in the “Statistical analysis” section on page 15: *“Socioeconomic status will be assessed at community and individual levels. Area-level disadvantage will be measured using the Australian Bureau of Statistics Socio-Economic Indexes for Areas (SEIFA) Index of Relative Socio-economic Disadvantage (IRSD) quintiles derived from participants’ residential postcode at recruitment. Individual-level socioeconomic characteristics, including educational attainment, employment status, household income and housing (collected via questionnaires), will also be used.”*

Comment 20: **If no subgroup and sensitivity analyses are planned, this should be stated.**
Response: We thank the reviewer for this valuable suggestion. Please see below responses:
<u>Subgroup analysis</u>
Subgroup analysis based on baseline inhaled corticosteroid (ICS) use, remoteness (regional vs metropolitan), body mass index (BMI) category and antenatal model of care are pre-specified and will be reported. Interaction terms will be used to aid interpretation of effect modification, recognising the limited power of such tests. This is now included in the statistical analysis section of the manuscript page16-17: *“Prespecified subgroup analyses will be conducted to explore potential effect modification by baseline inhaled corticosteroid (ICS) use, remoteness (regional versus metropolitan sites), body mass index (BMI) category and antenatal model of care. These subgroup analyses are hypothesis-driven and will be reported irrespective of statistical significance. Effect modification will be assessed using interaction terms included in multivariable models. Interaction p values will be interpreted cautiously, with emphasis on effect sizes and confidence intervals, recognising that interaction tests may be underpowered.”*
<u>Sensitivity analysis</u>
Sensitivity analysis will be conducted to assess measurement bias, as the primary study site in Newcastle will collect more detailed assessments of several traits in addition to the core measurements taken at all sites, this is now included in the “Statistical analysis” section on page 17: *“Sensitivity analyses will be conducted to assess the robustness of study findings. As the primary study site in Newcastle collects more detailed assessments of selected traits in addition to core measurements obtained at all sites, sensitivity analyses will be restricted to Newcastle participants. This will allow assessment of potential measurement bias by comparing results derived*

from core measurements across all sites with those based on the more detailed assessments in Newcastle."

***Comment 21:* How will missing data and 'loss to follow up' be accounted?**
Response: Thank you for this helpful comment. As attrition is expected to be low (10–15%) due to the short study timeframe, our primary analyses will use complete cases under a missing completely at random (MCAR) assumption. To address potential bias due to missingness, we will additionally conduct sensitivity analyses using multiple imputation under a MAR assumption for key outcomes and exposures. This detail has been added to the "Statistical analysis" section of the manuscript page 17.
"Missing data will be handled using complete case methods for the primary analyses, given the low anticipated level of attrition (10-15%). Sensitivity analyses will be conducted using multiple imputation under a missing at random (MAR) assumption for key outcomes and analyses. Imputed datasets will be generated using chained equations incorporating all variables included in the analysis models."

Comments on Discussion

***Comment 22:* A short description of how the associations explored in this study will improve care is needed to complete this paper.**
Response: Thank you for this helpful suggestion. The following text has been added to the last paragraph of the introduction on page 5 in keeping with journal formatting guidelines.
"This study will provide insight into the prevalence of all treatable traits in pregnancy, and their association with exacerbation risk, poor asthma control, and asthma-related quality of life. Filling these knowledge gaps will inform the feasibility of a treatable trait-based precision medicine approach for asthma management in the antenatal care setting and support the development of a more refined treatable trait focused management strategy. Interventions informed by this study have the potential to improve asthma outcomes during pregnancy as well as longer-term health outcomes for offspring."

***Comment 23:* The strengths and weaknesses of the study design should also be explored. The detailed examination of biomarkers should contribute unique insights and is an important strength. Some of the serious limitations are mentioned in the summary but need greater exploration. What strategies will be deployed to reduce volunteer selection bias? How will population representivity be checked? Collider bias is an important threat to volunteer cohorts. How will this be minimised? Which of the traits identified are mediators or moderators? How will this be accommodated in the analyses?**
Response: Thank you for these comments and thoughts. See below responses

Strengths and limitations
Strengths and limitations have been addressed in accordance with journal formatting requirements, with the addition of biomarkers to the first bullet point on page 3:
"with objective biomarkers strengthening clinical assessment beyond symptoms alone"
Further information has been added to the "Recruitment" section (page 7) and "Data analysis" section (page 13-17) describing approaches to minimise potential limitations.

Strategies to reduce volunteer selection bias
We recognise that volunteer studies are inherently susceptible to selection bias. To mitigate this, several design features have been incorporated to minimise participation barriers and reduce differential selection. These include flexible visit timing and alignment with routine antenatal appointments where possible, online questionnaires that can be completed remotely, the option of telehealth visits if women are unable to attend visits in person, and reimbursement for participant travel and time, the following text has been added to the "Data collection" section of the manuscript on page 8.
"To minimise participant barriers, most questionnaires can be completed remotely, participants will be reimbursed for their travel and time, they will be offered flexible visit timing and visits will be aligned with routine antenatal appointments where possible."

Importantly, recruitment pathways were deliberately diversified to reduce reliance on any single pathway that might preferentially select for disease severity, socioeconomic status and antenatal models of care. These pathways include:

1. Clinical referral to an asthma nurse
2. Community self-volunteer
3. Identification via electronic medical records with opt-out consent, which allows inclusion of women who may be less engaged with specialist asthma services.

This has been addressed in the final paragraph of recruitment in the manuscript page 7:
"These diverse recruitment pathways allow for inclusion of participants of all disease severity, socioeconomic status and antenatal model of care."

Assessment of population representativeness
Population representativeness will be evaluated by comparing key maternal demographic and antenatal characteristics with publicly available national and state perinatal datasets. These comparisons will allow assessment of the extent and direction of any selection effects and will be reported to aid interpretation of generalisability. This strategy has now been included in the "Statistical analysis" section on page 17:
"To assess external validity, cohort representativeness will be assessed by comparing key maternal demographic and antenatal characteristics with publicly available national and state perinatal data from the Australian Institute of Health and Welfare (AIHW) and Perinatal Data Collections from NSW and Victoria."

Collider bias and interpretation of associations

We acknowledge that conditioning on study participation in volunteer cohorts may introduce collider bias, particularly where participation may be influenced by both asthma severity and factors such as health literacy or healthcare engagement. Although such bias cannot be fully eliminated in an observational cohort design, we have taken several steps to minimise its impact. Broad recruitment pathways and opt-out identification aim to reduce conditioning on healthcare engagement alone. In the analysis phase, causal diagrams (directed acyclic graphs) will be used to explicitly consider selection mechanisms and avoid inappropriate adjustment for variables that may act as colliders or lie on causal pathways. Findings will be interpreted as associations rather than causal effects, with acknowledgement of residual selection and collider bias as a study limitation.

In the limitations on page 3 we have added *"eg asthma severity and health literacy"* to the limitation on selection bias. We have also added *"and we acknowledge there may be some residual selection and collider bias"* to the limitation on causal interpretation on page 3.

Mediators, moderators and analytical treatment of traits

The treatable traits framework includes variables that may plausibly operate as confounders, mediators, or moderators depending on the exposure-outcome relationship being assessed. Given the close temporal overlap of trait measurement during pregnancy and asthma outcomes later in pregnancy, formal mediation analyses are not planned. This will avoid misclassification of temporal ordering and bias from adjustment for intermediate variables. Instead, the focus will be on estimating associations and identifying potential effect modification. Pre-specified subgroup analyses will be treated as moderation analyses, assessed using interaction terms and stratified estimates informed by causal diagrams, this has been addressed in page 16 of Statistical Analysis: *"Prespecified subgroup analyses will be conducted to explore potential effect modification by baseline inhaled corticosteroid (ICS) use, remoteness (regional versus metropolitan sites), body mass index (BMI) category and antenatal model of care. These subgroup analyses are hypothesis-driven and will be reported irrespective of statistical significance. Effect modification will be assessed using interaction terms included in multivariable models. Interaction p values will be interpreted cautiously, with emphasis on effect sizes and confidence intervals, recognising that interaction tests may be underpowered."*

Confounder selection will be tailored to each exposure-outcome pair, to avoid inappropriate adjustment, this has been added to the "Statistical analysis" section page 15: *"Directed acyclic graphs (DAGs) will be used to identify potential confounders and minimal adjustment sets for each exposure and outcome model. The main confounding set will include demographic variables (socioeconomic status at community and individual levels, maternal age, remoteness, BMI) and pregnancy-related characteristics, including antenatal model of care, gestational weight gain, relevant*

environmental exposures, smoking during pregnancy (except in models where smoking is the trait of interest) and other treatable traits as relevant. An example DAG can be found in the supplement (Figure S1).”

General Comments

Comment 24: **References might usefully be updated.**

Response: We thank the reviewer for this comment. While we considered updating the references, we believe the current citations remain the most relevant and appropriate for the aims and scope of this manuscript.

Comment 25: **I did not find a reporting guideline. The EQUATOR website recommends the STROBE checklist for protocols for observational studies.**

Response: We have now included a STROBE checklist in the supplementary material (Table S10).

Comment 26: **Is the study registered with a database?**

Response: As this is an observational study only it is not registered with a database.

Comment 27: **Ethical approvals should be dated.**

Response: These dates have now been included in the manuscript under the ethics and governance section (page 18).

Comment 28: **The responsible statistician should be named.**

Response: The responsible statistician is now named in the “Authors’ contributions” section page 20.

Comments on Language

Comment 29: **‘Long-term conditions’ might be considered less pejorative than ‘common chronic diseases’.**

Response: Thank you for this feedback, this wording has now been changed to *“prevalent long-term health conditions”* on pages 2 and 4.

Comment 30: **I suggest all summary bullet points are presented as sentences.**

Response: Thank you for this feedback. The bullet points in the “Strengths and Limitations” section are consistent with the BMJ Open author guidelines for protocol papers so have not been changed.

Comment 31: **I noted grammatical errors: e.g. p.7 line31/32; p.8 line22; p.12 line 4; p.19 line 30.**

Response: Thank you for these observations. These errors have been corrected. Paragraph 4 of introduction (page 5), paragraph 1 of inclusion criteria (page 6),

paragraph 3 of biological samples (page 11) and heading for authors' contributions (page 20).

Comment 32: **Some abbreviations were not evident in the table foot notes.**
Response: Thank you for bringing this to our attention. These abbreviations have now been added to the footnotes of Table 1(pages 13-14).

VERSION 2 - REVIEW

**Reviewer**	**2**
**Name**	**Jordan, Sue**
**Affiliation**	**Swansea University, Nursing**
**Date**	**03-May-2026**
**COI**	

Thank you for the opportunity to read this re-submitted protocol. The points raised have now been addressed.
